# Similar trends of susceptibility in *Anopheles arabiensis* and *Anopheles pharoensis* to *Plasmodium vivax* infection in Ethiopia

**DOI:** 10.1186/s13071-016-1839-0

**Published:** 2016-10-18

**Authors:** Nuredin Abduselam, Ahmed Zeynudin, Nicole Berens-Riha, Dinberu Seyoum, Michael Pritsch, Habtewold Tibebu, Kasahun Eba, Michael Hoelscher, Andreas Wieser, Delenasaw Yewhalaw

**Affiliations:** 1 Department of Medical Laboratory Sciences and Pathology, College of Public Health and Medical Science, Jimma University, Jimma, Ethiopia; 2 Department of Bacteriology, Max von Pettenkofer-Institute (LMU), Munich, Germany; 3 Division of Infectious Diseases and Tropical Medicine, Medical Center of the University of Munich (LMU), Munich, Germany; 4 Department of Statistics, Natural Science College, Jimma University, Jimma, Ethiopia; 5 German Center for Infection Research (DZIF), partner site Munich, Munich, Germany; 6 Division of Cell and Molecular Biology, Department of Life Sciences, Imperial College London, London, United Kingdom; 7 Tropical and Infectious Diseases Research Center, Jimma University, Jimma, Ethiopia; 8 Institute of Health and Society (IRSS), Université catholique de Louvain, Brussels, Belgium

**Keywords:** Experimental infection, *Anopheles arabiensis*, *Anopheles pharoensis*, *Anopheles coustani*, *Plasmodium vivax*, Direct membrane feeding assay, Malaria

## Abstract

**Background:**

Around half of the global population is living in areas at risk of malaria infection. *Plasmodium vivax* malaria has become increasingly prevalent and responsible for a high health and socio-economic burden in Ethiopia. The availability of gametocyte carriers and mosquito species susceptible to *P. vivax* infection are vital for malaria transmission. Determining the susceptibility of vector species to parasite infection in space and time is important in vector control programs. This study assesses the susceptibility of *Anopheles arabiensis*, *An. pharoensis* and *An. coustani* group to *Plasmodium vivax* infection in Ethiopia.

**Methods:**

Larvae of *An. arabiensis*, *An. pharoensis* and *An. coustani* group were collected from an array of breeding sites and reared to adult under controlled conditions. Batches of adult female mosquitoes of the three species were allowed to feed in parallel on the same infected blood with gametocytes drawn from *Plasmodium vivax* infected patients by Direct Membrane Feeding Assays (DMFA). Fed mosquitoes were kept in an incubator under controlled laboratory conditions. Seven days after each feeding assay, mosquitoes were dissected for midgut oocyst microscopy and enumeration. Data were analysed using R statistical software package version 3.1.0.

**Results:**

Over all, 8,139 adult female mosquitoes were exposed to *P. vivax* infection. Of the exposed mosquitoes 16.64 % (95 % CI: 1,354–8,139) were properly fed and survived until dissection. The infection rate in *An. arabiensis* and *An. pharoensis* was 31.72 % (95 % CI: 28.35–35.08) and 28.80 % (95 % CI: 25.31–32.28), respectively. The intensity of infection for *An. arabiensis* and *An. pharoensis* was 2.5 (95 % CI: 1.9–3.2) and 1.4 (95 % CI: 1.1–1.8), respectively. Gametocyte density was positively correlated to infection rate and intensity of infection in *An. arabiensis* as well as *An. pharoensis*. No *An. coustani* group mosquitoes were found infected, though almost four hundred mosquitoes were successfully fed and dissected. All groups received blood from the same infected blood source containing gametocytes in parallel. There was no significant difference in susceptibility rates between *An. arabiensis* and *An. pharoensis* (*P* = 0.215).

**Conclusions:**

*Anopheles arabiensis* and *An. pharoensis* showed similar susceptibility to *P. vivax* infection. However, *An. coustani* group was not permissive for the development of *P. vivax* parasites.

## Background

Malaria is the most widespread mosquito-borne disease posing a potential health risk to almost half of the world’s population. Around 3.2 billion people, living in 106 countries with ongoing transmission, are at risk of active malaria infection. In 2015, around 214 million malaria cases and 438,000 deaths were reported globally [1]. *Plasmodium vivax* malaria is not common in Africa except for Ethiopia, and most disease cases are attributable to *P. falciparum*, which is responsible for 90 % of malaria related deaths. Outside Africa, *P. falciparum* and *P. vivax* almost invariably coexist and are often equally prevalent, while the former is the most important public health threat [2]. *Plasmodium vivax* is the most cosmopolitan of all malaria parasites, reaching latitudinal extremes of 64 °N and 32 °S [3].

*Plasmodium vivax* is common in East Asia, Western Pacific, Central and South America, and is responsible for many millions of annual cases of malaria [4]. *Plasmodium vivax* threatens almost 40 % of world’s population where south-east Asia alone accounts for 67 % of global cases [5]. In 2013, *P. vivax* was estimated to be accountable for 16 million malaria cases in the world, and almost half of the disease cases outside of Africa. *Plasmodium vivax* causes significant morbidity and mortality, and is recognized to be a major barrier to achieving the targets set in the malaria elimination and eradication strategies. Besides, it is ever more recognized that *P. vivax* malaria disease can be as severe and deadly as *P. falciparum* malaria [5].

Ethiopia is one of the most malaria affected countries where 70 % of the country’s population live in active malaria transmission areas, and are constantly at risk of infection. Except for the central highlands of the country, which are malaria free, malaria is a serious problem [6]. In Ethiopia, malaria disease is caused by four *Plasmodium* spp. where *P. falciparum* and *P. vivax* are almost equally prevalent and accountable for 60 % and 40 % of the cases, respectively. *Plasmodium malariae* and *P. ovale* are only responsible for less than 1 % of the total cases [7].

The prevalence of malaria disease is variable with seasons and in different regions of the country [8]. In south-central Ethiopia, 86.5 % of the cases are attributable to *P. vivax* malaria whereas *P. falciparum* is only accountable for 12.4 % [9]. The intensity of malaria disease is unstable and usually occurs as widespread epidemics at intermittent time intervals [10]. Malaria transmission is mostly influenced by the composition of susceptible mosquito species, the vectorial capacity and environmental factors such as, topography, rainfall, local environmental conditions as well as socioeconomic and demographic variables of the population at risk [11].

Around 43 *Anopheles* mosquito species are documented in Ethiopia; however, only a few species are incriminated as vectors of malaria [12]. *Anopheles gambiae* (*s.l*.), the most important vectors of malaria [13], comprises of eight sibling species that are morphologically indistinguishable but can be differentiated with their specific behavior, ecology, relative frequency, distributions and vector competence [14, 15]. Populations of *An. arabiensis*, *An. pharoensis* and *An. coustani* usually co-occur in south-western Ethiopia [16]. *Anopheles arabiensis* and *An. pharoensis* populations occur in abundance during the rainy seasons in some parts of Ethiopia, whereas *An. pharoensis* population is significantly higher in abundance than that of *An. arabiensis* in dry seasons [17].

*Anopheles arabiensis* is adapted to diverse ecological conditions, flexible feeding preference, seasonal occurrence and high vectorial capacity. *Anopheles arabiensis* is the principal vector of malaria in Ethiopia which is adapted to diverse, spatial and temporal malaria transmission patterns [18]. *Anopheles funestus*, *An. nili* and *An. pharoensis* are incriminated as the secondary vectors of malaria in the country [19]. *Anopheles arabiensis* and *An. pharoensis* together are important vectors of *P. vivax* as well as *P. falciparum* malaria in the low and mid altitude areas of Ethiopia [20]. In south-central Ethiopia, malaria transmission during the dry season is supported by *An. arabiensis* and *An. pharoensis* due to the land use practices favoring mosquitoes breeding in this area [17, 21].

Since the past decade, due to scaled up interventions, malaria-related morbidity and mortality declined in high-burden countries, like Ethiopia. Malaria control interventions have decreased the number of cases and related deaths in Ethiopia [22]. However, malaria still remains a major public problem [5]. The decline in the burden of malaria is associated with the overall decline in the density of vector population [23]. The most widely used vector control methods, long-lasting insecticidal nets (LLINs) and indoor residual spraying (IRS), are employed to reduce vector-human contact targeting on the most highly anthropophagic as well as endophilic adult mosquito species [24]. *Anopheles gambiae* (*s.s*.) and *An. funestus* are highly anthropophilic and rest indoors; and so malaria control experts have continued to design and distribute vector control interventions that can appropriately tackle those vector species which are responsible for most of the indoor malaria transmission [25].

Vector control interventions have affected individual mosquito species biting behavior and thus resulted in shifts in effective vector composition [26]. The decrease in the density of the vector population is mostly marked for *An. gambiae* (*s.s*.) and *An. funestus*, but least for *An. arabiensis* and *An. pharoensis* [27]. The *An. arabiensis* population has changed its position from being the rarest one in the past to the most common in the present [28]. This may be linked to changed resting- and host selection behaviors by switching to feed on cattle or rest outdoors avoiding contact to insecticide sprayed surfaces and also showing an adaptive shifting from strongly indoor to outdoor resting and host seeking behavior [28–30], thus compromising vector control strategies [5]. Especially *An. arabiensis* is adapted to flexible feeding preferences, shows seasonal occurrence and high vectorial capacity, enabling a diverse, spatial and temporal malaria transmission pattern. Although the highest research attention has been given to *An. gambiae* (*s.s*.), because of the high degree of anthropophilic behavior, vectorial capacity and sequenced genome [31], its position is replaced by *An. arabiensis* as the major vector of malaria in Africa. The secondary vectors of malaria have been considered as unimportant in the past; however, the role of secondary vectors in malaria transmission has significantly increased over the years [32]. The role of *An. coustani* complex in East Africa is still unclear.

The difference in susceptibility to infection among malaria vector species is usually determined by comparing their natural infection rates; which is usually determined by the prevalence of infections in wild-caught adult mosquitoes. Despite the differences in susceptibility, the chance of mosquito’s infection is affected by various environmental, biological as well as behavioral factors. The susceptibility of *An. arabiensis* to *P. vivax* infection, in comparison to *An. pharoensis*, has not been experimentally determined in Ethiopia. A study conducted in Kenya documented that *An. coustani* is susceptible to malaria infection and reported to be vector of malaria in the area [32]. However, its susceptibility to malaria infection has not yet been experimentally verified in Ethiopia.

The aim of the present study was to determine the comparative physiological susceptibility to *P. vivax* infection in *Anopheles arabiensis*, *An. pharoensis* and *An. coustani* group in Jimma, Ethiopia. The experiments were carried out with batches of mosquitoes of the three different mosquito species membrane-fed with freshly drawn *P. vivax* infected blood from naturally infected gametocyte carriers under controlled laboratory conditions.

## Methods

### Study site

The study was conducted in Jimma town, south-west Ethiopia (7°41'N, 36°50'E). Jimma is located 1,780 meters above sea level in the western zone of cool tropical climate. It is located 335 km south-west of the capital, Addis Ababa. Annual minimum and maximum temperatures ranges from 14 °C to 30 °C, respectively. The mean annual rainfall ranges from 1,138 to 1,690 mm. The minimum and maximum precipitations occur during the dry and wet season, respectively. The area is malaria endemic with moderate transmission and the principal malaria parasites are *P. falciparum* and *P. vivax*. Populations of *An. arabiensis*, *An. pharoensis* and *An. coustani* group are sympatric in most areas in Jimma. There are naturally infected *P. vivax* gametocyte carriers in the population*.* Experimental infection data were collected from June to October, 2014.

### Mosquito rearing and identification

Mosquito samples were collected as adult stages and as late stage larval instars in Bore Tika village, 5 km south of Jimma town, south-western Ethiopia. Adult mosquitoes were collected from resting sites in human dwellings and from animal sheds using enclosure traps as well as mouth aspirators from 7 pm to 8 pm and 5 am to 6 am. All adult mosquitoes were morphologically identified to the species level using taxonomic keys [33]. Adult female *An. gambiae*, *An. pharoensis* and *An. coustani* group mosquitoes were then transported to Jimma University experimental insectary in paper cups sealed with mesh material. All *An. gambiae* mosquitoes in Jima zone are *An. arabiensis* [34, 35]. This finding is confirmed by unpublished studies performed previously by our group as well. Unfed female mosquitoes were separately placed in paper cups and allowed to feed on blood using membrane feeders. Fed mosquitoes of each species were kept separately in different cages with a daily provision of 10 % sucrose solution. Gravid mosquitoes were soon transferred for oviposition and further steps of the rearing processes were performed following standard procedures [36].

Larvae were collected from natural breeding sites using standard dipping method. The extent of larval sampling per breeding site was limited in order to avoid bias from oversampling of the sibling species in *An. coustani* group. Mosquito larvae were immediately filtered so as to avoid larvae predators, competitors and unwanted debris, transported to the insectary in water taken from the mosquito natural breeding sites, and reared to adult stages on yeast and dog biscuits. Emerging adult females were maintained in cages with 10 % sucrose solution. Two to three day-old female adult mosquitoes were pooled in cups and kept for experimental infection.

### Screening of gametocyte carriers

Gametocyte carriers were screened from patients visiting Jimma Shenen Gibe Hospital. *Plasmodium vivax* infected patients were screened after microscopic examination and gametocyte detection by standard finger prick and Giemsa stained blood smears. Parasite density was determined for 100 microscopic fields of the blood smear. The asexual and sexual parasite stages were counted against 200 and 500 leukocytes, respectively. Parasite density was determined against 8,000 leukocytes/μl of blood. Screening of gametocyte carriers was synchronized to mosquito rearing processes and thus always performed on the same day with the experimental infection.

### Direct membrane feeding assay (DMFA)

An artificial membrane feeding apparatus with 10 micro-glass feeders of equal volume (2 cm diameter each), was used for experimental mosquito infection. These systematically connected glass feeders enabled to feed in parallel up to 400 mosquitoes at a time. Thus, all membrane closed glass feeders were placed on top of the paper cups with starved mosquitoes. During this parallel feeding process, temperature was maintained at 37 °C (± 0.1 °C) using a water jacket of high speed circulation system [37].

In brief, a temperature controlled water bath with a pump (Model 8005, Polyscienca, Illinois, USA) maintained at 37 °C was used to pump warm water through the miniature glass feeders in parallel. The temperature in all feeders was controlled and found to be at the same temperature of ± 0.1 °C among the ten individual glass feeders. The feeders were closed at the bottom using pre-streched Parafilm (National Can, Chicago, USA) and filled with 100 μl of freshly drawn blood [37]. The micro-feeders were kept on the paper cups with starved mosquitoes covered with mesh and allowed to feed simultaneously on the same infected blood (same gametocyte source) for a period of 20 min under calm and reduced light conditions. Subsequently, the glass feeders were removed and unfed mosquitoes were removed from the cups and killed. Fed mosquitoes were maintained in a climate chamber set at 27 ± 1 °C and 75 ± 5 % relative humidity (EMKO ESM-4450, Mytron, Heilbad Heiligenstadt, Germany). Mosquitoes were provided with cotton balls soaked with 10 % sucrose solution daily.

### Mosquito dissection

Seven days post-feeding, all surviving mosquitoes were immobilized using CO_2_ and dissected under stereo microscopes for oocyst examination and enumeration. Midguts were removed and stained with 2 % mercurochrome solution in phosphate–buffered saline (PBS). Midgut oocysts were examined and counted using a microscope at 400-fold magnification using a 40× objective. For each of the 20 infection sessions, the infection rate (IR) (infected mosquitoes/total mosquitoes dissected in individual feeding, given in percent), and intensity of infection (II) (mean oocyst burden/total dissected mosquitoes in individual feeding) were determined. Non-infected mosquitoes were removed from the calculation.

### Data analysis

Data were analyzed using R statistical software package version 3.1.0. The rate of susceptibility to infection was compared among the three mosquito species. Two parameters were chosen to compare the rate of susceptibility to *P. vivax* infection between populations of *An. arabiensis* and *An. pharoensis*. IR and II in dissected mosquitoes were computed for each species. The overall IR and II were compared between populations of *An. arabiensis* and *An. pharoensis* using Pearson's Chi-square test and Wilcoxon or Mann-Whitney tests, respectively. The correlations of blood gametocyte density IR and II were also assessed for *An. arabiensis* and *An. pharoensis* using . A box-plot was generated to visualize median values of covariates. Statistical significance for association of variables, mean comparisons and confidence intervals were considered at *P* < 0.05.

## Results

Of the total of 471 febrile subjects screened for malaria infection via blood film examination, 108 (23 %) were confirmed to be malaria positive. Of these, 63 (58 %) were found to be infected with *P. vivax* and 33 (30.5 %) were microscopically identified as *P. vivax* gametocyte carriers. Although 24 gametocyte carrier individuals fulfilled the inclusion criteria and enrolled in the experimental infection, only 20 successful mosquito feeding assays were considered during the analysis. Blood gametocyte density among gametocyte carriers was ranging from 64 to 912 gametocytes/μl. The mean asexual and sexual parasite density was 5,974/μl and 367/μl, respectively. In the 20 independent experimental infections, 8,139 female mosquitoes from the three species were used in the feeding experiments. Of these, 3,583 (44 %) *An. arabiensis*, 2,202 (27.05 %) *An. pharoensis*, and 2,354 (28.92 %) *An. coustani* group were exposed to infected blood. Before each infection session, each mosquito batch was kept separately in paper cups and starved for 24–36 h. For every single infection session, nearly 400 adult mosquitoes were used. During each infection session, 35–40 starved mosquitoes were fed with the infected blood per paper cup. Of the total 8,139 mosquitoes allowed to feed on infected blood, 1,689 (20.75 %) fed successfully in the 20 independent experimental infections. Feeding efficiency for *An. arabiensis*, *An. pharoensis* and *An. coustani* group was comparable with 19.8 %*,* 22.43 % and 20.56 %, respectively. In contrast, 1,041 (12.8 %) exposed mosquitoes died before feeding on the infected blood. Mosquito mortality rate for *An. arabiensis*, *An. pharoensis* and *An. coustani* group prior to feeding on the infected blood was also similar between the species with 11.6 %, 13 % and 14.3 %, respectively (Table 1). Moreover, of the 1,689 fed mosquitoes, 335 (19.8 %) died during incubation and were removed, while the remaining 1,354 (80.1 %) mosquitoes survived to day 7, post-feeding and were brought to dissection. Successful dissection with sufficient microscopic preparation of the midgut could be obtained in 1,318 mosquitoes, of which 579 (43 %) belonged to *An. arabiensis*, 402 (30.5 %) belonged to *An. pharoensis* and 373 (25.5 %) belonged to *An. coustani* group*.* On average, 66 mosquitoes were dissected per infection session, and all dissected mosquitoes were considered during the analysis.

**Table 1:** Anopheline mosquito species by their physiological state and infectivity rates following feeding assay in Jimma, South-Western Ethiopia

Mosquito feeding status	*An. arabiensis*	*An. pharoensis*	*An. coustani* group
Dead before feeding	418	286	337
Unfed	2,454	1,422	1,533
Fed and incubated	711	494	484
Dead before day 7	132	92	111
Survived to day 7	579	402	373
Infected guts	185 (31.95 %)	116 (28.86 %)	0 (no infection)
Total exposed	3,583	2,202	2,354

On average, 301 (22.2 %) mosquitoes were infected with microscopically detectable oocysts. Of 579 *An. arabiensis* and 402 *An. pharoensis* dissected, 185 (31.95 %) and 116 (28.86 %) of *An. arabiensis* and *An. pharoensis* were infected, respectively. However, of the 373 fed and dissected *An. coustani* group, no single individual mosquito was found to be infected (Table 1).

**Fig. 1:**
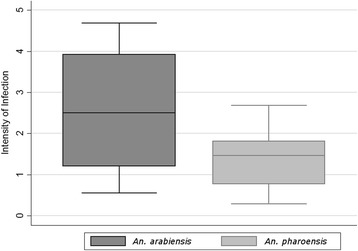
Box plot of the mean intensity of infection of *P. vivax* measured with direct membrane feeds in the populations of *An. arabiensis* and *An. pharoensis*

Mean IR for *An. arabiensis* and *An. pharoensis* was 31.72 % (95 % CI: 28.35–35.08) and 27.80 % (95 % CI: 25.31–32.28), respectively (Fig. 1). Overall pairwise comparison of IR between *An. arabiensis* and *An. pharoensis* was similar (*χ* ^2^ = 38.00, *df* = 32, *P* = 0.215). In contrast, overall pairwise comparison revealed that the difference in mean II between *An. arabiensis* and *An. pharoensis* was significant (*Z* = -2.651, *P* = 0.008). The maximum oocyst load counted in the midgut of individual infected *An. arabiensis* and *An. pharoensis* was 16 and 12, respectively. Moreover, the mean II for populations of *An. arabiensis* and *An. pharoensis* was 2.5 (95 % CI: 1.9–3.2) and 1.4 (95 % CI: 1.1–1.8), respectively. Plasmodia infection was absent in all dissected *An. coustani* mosquitoes.

**Fig. 2:**
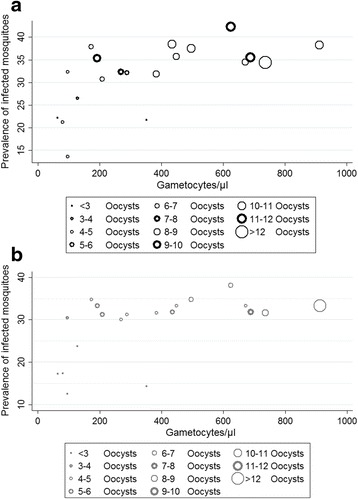
Correlation between *Plasmodium vivax* gametocyte densities in blood (x-axis) and infection rates. Individual feeds are presented in different diameters depending on the average number of detected oocysts in infected dissected mosquitoes (II). DMFA performed in parallel with *An. arabiensis* (**a**) and *An. pharoensis* (**b**)

**Fig. 3:**
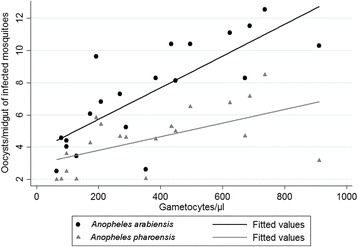
Correlation between intensity of infection and gametocyte density/μl of blood in populations of *An. arabiensis* (*r =* 0.79, *P* < 0.001) and *An. pharoensis* (*r =* 0.59, *P* = 0.006). No infected *An. coustani* group mosquitoes detected in the performed DMFA

A significant positive correlation between gametocyte density and IR was also recorded for both *An. arabiensis* (*r =* 0.61, *P* = 0.004) and *An. pharoensis* (*r =* 0.54, *P* = 0.015). However, there was variability in the association between gametocyte density and IR for the two species (Fig. 2a, b). Gametocyte density and II was also significantly correlated in *An. arabiensis* (*r =* 0.79, *P* < 0.001) and *An. pharoensis* (*r =* 0.59, *P* = 0.006). Although there was a variation in II with gametocyte density between individual infection sessions there was an overall increasing trend of II with gametocyte load in *An. arabiensis* and in *An. pharoensis* (Fig. 3).

## Discussion

This is the first study assessing the susceptibility of malaria vectors to *P. vivax* infection using membrane feeding assays in Ethiopia. Experimental mosquito infection through DMFA using freshly drawn human blood is one of the challenging experimental setups in malaria research. In the present study midgut oocysts developed efficiently in *An. arabiensis* and *An. pharoensis* and there was a similar trend of infection in both species after feeding on gametocytes in *P. vivax* infected blood. Despite *An. coustani* is known to play important roles in *P. falciparum* transmission in Kenya [32], in this study, plasmodia infection was absent in all 373 *An. coustani* mosquitoes that were fed with infected blood.

The difference in susceptibility to malaria parasite infection in *Anopheles* mosquito species could be attributed to parasite mortality during penetration of gut wall or due to mosquito’s immune response, as observed in *P. berghei* [38]. Since melanized midgut oocysts were not detected microscopically in this study, the possible reason why *An. coustani* group did not support the development of *P. vivax* parasites remains unclear. The mosquito’s refractoriness could be attributed to the immune status of the patients; or it might also be attributed to the biological reactions in mosquito’s innate immune system, or the mosquitoes gut flora properties, leading to lysis or melanisation of midgut ookinetes [39]. Mosquitoes of *An. coustani* group might have complete refractoriness against parasite development in the midgut resulting in the complete clearance of ookinetes.

Based on the natural infection rates, entomological inoculation rates (EIR) and other parameters of vectorial capacity, *An. arabiensis* and *An. pharoensis* are recognized as the principal and secondary vectors of malaria*,* respectively, in Ethiopia. The vectors natural infection rate is commonly determined based on prevalence of plasmodia infections in wild caught adult mosquitoes. However, mosquito’s natural infection rates are affected by various environmental factors, behavioral patterns and species-specific physiological susceptibility. The EIR of *Anopheles* mosquitoes depends on human biting frequencies and physiological susceptibility to gametocytes infection [40].

In the current study, the species-based physiological susceptibility to gametocyte infection was determined in *An. arabiensis* and *An. pharoensis* mosquito populations under controlled environmental conditions. Species-specific physiological susceptibility differences in *Anopheles* mosquitoes might have resulted in different levels of IR or II. IR and II together or individually were considered as indicators of mosquito’s infection and thus used to compare the level of physiological susceptibility between *An. arabiensis* and *An. pharoensis* to malaria parasite infection. As shown in the results of the study, the overall pair wise comparison demonstrated a significant difference in infection intensities between *An. arabiensis* and *An. pharoensis* despite the parallel nature of the exposure.

The detection of oocysts in the midgut seven days following blood meal is a standard method to assess susceptibility to parasite infection in mosquitoes; however, this might not accurately evaluate the infectiousness of mosquitoes to human hosts [41]. In experimental infections, not all oocysts are equally expected to effectively contribute to mosquito’s infectiousness. The impact of oocyst arrest in low intensity infections could lead to failure in sporozoites release into the haemocoel; consequently, this could affect the reliability of oocyst prevalence as a measure of infectivity in mosquitoes [42]. Though the variation in oocyst load between *An. arabiensis* and *An. pharoensis* was significant, infected mosquitoes were not maintained for more than 7 days after the blood meal and the difference in efficiency of sporozoite development was not assessed during the experimental study.

In experimental infections of *An. gambiae* (*s.s*.) with *P. falciparum* most infected mosquitoes in the field had very low midgut oocyst load but supported the development of the parasite to the sporozoite stage. Furthermore, the majority of oocyst positive mosquitoes in infection studies may have at least one ruptured oocyst [43] from which sporozoites reach the salivary gland. During mosquito’s blood meal, the number of salivary sporozoites injected and required to infect a human host (< 100) is considerably less than the number of sporozoites often produced by one oocyst (range 1,359–14,000) indicating that a single oocyst can be sufficient to make a mosquito infectious [44].

The correlation between gametocyte density and prevalence as well as intensity of infection in mosquitoes, were not similar throughout the infection sessions, which might be attributed to the presence of confounding factors which increased its variability. It is to be noted, that some feeding experiments with relatively higher blood gametocyte load did not result in higher infection in both mosquito species, whereas others with low gametocytemia resulted in higher infection. The variations in infectivity could be due to differences in the immune status of the patients and density of matured gametocytes in the blood samples.

Despite the differences in mosquito species and parasite genome, our findings were in line with the findings of the study from Brazil where *P. vivax* gametocyte density was significantly correlated to infectivity rates in wild mosquitoes [45]*.* However, it is not in agreement with the findings reported from China where the parasite load was significantly correlated to IR; but not with II in laboratory colonized *An. sinensis* [46]. Although gametocyte densities were determined microscopically, and only patients with visible gametocytemia were used for infection experiments, the immunological status of the patients, anti-malaria antibodies, might have influenced the infection rate which might be responsible for the variability in infection [47]. Moreover, handling of freshly drawn blood might also trigger the exflagellation of microgametes and thus reduce oocyst formation as this might not be completely excluded, although strict standard operation procedures (SOP) were followed for each step during the experiment.

Although the mosquitoes from the three species were fed in parallel on the same source of gametocytes, the findings in the current study suggest that the population of *An. coustani* group is not physiologically permissive to oocyst development, suggesting that it may not have a role in transmitting *P. vivax* malaria at least in the Jimma area. However, it is to be noted that a formal exclusion may require infecting larger samples of mosquitoes obtained from a larger variety of regions. In contrast, populations of *An. arabiensis* and *An. pharoensis* were found to efficiently produce midgut oocysts after being fed from the same gametocyte source. Moreover, overall difference in IR between these two species was not significant. This shows that populations of *An. arabiensis* and *An. pharoensis* were almost equally susceptible to *P. vivax* infection.

Comparing from the perspective of infection intensity however, there was a significant difference between the two species. This difference in II between *An. arabiensis* and *An. pharoensis* could be due to difference in natural resting site preferences, which may indicate different behavioral patterns (e.g. *An. pharoensis* may not prefer to rest on walls longer as compared to the *An. arabiensis* population; this could lead to stress, deterring effects in the gut nutrient balance and finally to reduced fitness in midgut ookinetes). Estimating the proportion of infected mosquitoes after feeding on naturally infected blood with different gametocyte densities is the most relevant outcome measure to assess susceptibility to parasite infection in malaria vectors and to estimate the human reservoir of infection [47]. From oocyst prevalence, populations of *An. arabiensis* and *An. pharoensis* were found to be equally susceptible to *P. vivax* infection, whereas *An. coustani* was found to be refractory.

## Conclusions

*Anopheles arabiensis* and *An. pharoensis* were efficiently infected with *P. vivax* gametocytes, while *An. coustani* group was not infected. As dissections were only performed at seven days, post-feeding, no data were generated on sporozoite rate for *An. arabiensis* and *An. pharoensis*. The exact stage at which *P. vivax* development is blocked in the midgut of *An. coustani* was not determined in this study. The mechanisms leading to refractoriness of *An. coustani* group to *P. vivax* parasites development remain unclear. In the current study, the reliability and reproducibility of infections obtained in *An. arabiensis* and *An. pharoensis* demonstrated the efficiency of feeding protocols used in each independent experimental infection. As compared by the IR, *An. arabiensis* and *An. pharoensis* population were found to be equally susceptible to *P. vivax* infection. Therefore, given the ability of *P. vivax* parasites to establish infection in mosquito vectors at very low gametocyte density and little research attention given to secondary vectors, *An. pharoensis* could be a species that potentially poses a challenge against malaria elimination efforts in Ethiopia.

## Abbreviations

CI: Confidence interval
DMFA: Direct membrane feeding assay
II: Infection intensity
IR: Infection rate
IRS: Indoor residual spraying
LLIN: Long-lasting insecticidal net

## References

[1] WHO. World Malaria Report. Geneva: World Health Organization; 2015. http://www.who.int/entity/malaria/publications/world-malaria-report-2015/en/index.html. Accessed 13 Sept 2016.

[2] Hay SI, Guerra CA, Tatem AJ (2004). The global distribution and population at risk of malaria: past, present, and future. Lancet Infect Dis..

[3] Snow RW, Gilles HM. The epidemiology of malaria. In: Warrell DA, Gilles HM, editors. Essential malariology. 4th ed. London: Arnold; 2002. p. 85–106

[4] WHO (2010). World Malaria Report.

[5] WHO. Control and Elimination of *Plasmodium vivax* Malaria: World Health Organization, A Technical Brief. 2015. (http://apps.who.int/iris/bitstream/10665/181162/1/9789241509244_eng.pdf?ua=1). Accessed Sept 2016.

[6] Federal Democratic Ethiopia MoH. National Five-year Strategic Plan for Malaria Prevention and Control in Ethiopia. Addis Ababa: Minister of health; 2006.

[7] Fornadel CM, Norris LC, Norris DE (2010). Centers for disease control light traps for monitoring *An. arabiensis* human biting rates in an area with low vector density and high insecticide-treated bed net use. Am J Trop Med Hyg.

[8] Yewhalaw D, Legesse W, Bortel WV, Solomon G-S, Kloos H, Duchateau L, Peybroeck N (2009). Malaria and water resource development: the case of Gilgel-Gibe hydroelectric dam in Ethiopia. Malar J..

[9] Woyessa A, Deressa W, Ali A, Lindtjorn B (2012). Prevalence of malaria infection in Butajira area, south-central Ethiopia. Malar J..

[10] Abeku TA, van Oortmarssen GJ, Borsboom G, de Vlas SJ, Habbema JD (2003). Spatial and temporal variations of malaria epidemic risk in Ethiopia: factors involved and implications. Acta Trop..

[11] Kelly-Hope L, Hemingway J, McKenzie F (2009). Environmental factors associated with the malaria vectors *Anopheles gambiae* and *Anopheles funestus* in Kenya. Malar J..

[12] Coetzee M, Craig M, Sueur DL (2000). Distribution of African malaria mosquitoes belonging to *Anopheles gambiae* complex. Parasitol Today.

[13] Hunt RH, Coetzee M, Fettene M (1998). The *Anopheles gambiae* complex: a new species from Ethiopia. Trans R Soc Trop Med Hyg..

[14] Coetzee M, Hunt R, Wilkerso R, Torre A, Coulibaly M, Besansky N (2013). *Anopheles coluzzii* and *Anopheles amharicus*, new members of the *Anopheles gambiae* complex. Zootaxa..

[15] Lindsay SW, Parson L, Thomas CJ (1998). Mapping the ranges and relative abundance of the two principal African malaria vectors, *Anopheles gambiae s.s.* and *An. arabiensis*, using climate data. Proc R Ent Soc Lond.

[16] Degefa T, Ahmed Z, Ameyu G, Yohannes HM, Endalew Z, Daniel E (2015). Malaria incidence and assessment of entomological indices among resettled communities in Ethiopia: a longitudinal study. Malar J..

[17] Kibret S, Alemu Y, Boelee E, Tekie H, Alemu D, Petros B (2010). The impact of a small-scale irrigation scheme on malaria transmission in Ziway area, Central Ethiopia. Trop Med Int Health.

[18] White GB, Tessfaye F, Boreham PFL, Lemma G (1980). Malaria vector capacity of *Anopheles arabiensis* and *Anopheles quadriannulatus* in Ethiopia: chromosomal interpretation after 6 years storage of field preparations. Trans R Soc Trop Med Hyg..

[19] Krafsur ES, Armstrong JC. An integrated view of entomological and parasitological observations on *falciparum* malaria in Gambela, Western Ethiopian Lowlands. Trans R Soc Trop Med Hyg. 1978;72:348–56. 10.1016/0035-9203(78)90125-6 360497

[20] Animut A, Balkew M, Gebre-Michael T (2013). Blood meal sources and entomological inoculation rates of anophelines along a highland altitudinal transect in south-central Ethiopia. Malar J..

[21] Animut A, Gebre-Michael T, Balkew M, Lindtjørn B (2012). Abundance and dynamics of Anopheline larvae in highland malarious area of south-central Ethiopia. Parasit Vectors..

[22] Federal Democratic Ethiopia MoH. Ethiopia National Malaria Indicator Survey: Technical Summary. Addis Ababa: Mnister of health; 2012.

[23] Meyrowitsch DW, Pedersen EM, Alifrangis M, Scheike TH, Malecela MN, Magesa SM (2011). Is the current decline in malaria burden in sub-Saharan Africa due to a decrease in vector population?. Malar J..

[24] Federal Democratic Ethiopia MoH. National Malaria Strategic Plan 2014–2020. Addis Ababa: Minister of health; 2014.

[25] White GB, Magayuka SA, Boreham PFL (1972). Comparative studies on sibling species of the *Anopheles gambiae* Giles complex (Diptera: Culicidae): bionomics and vectorial activity of species A and species B at Segera, Tanzania. Bull Entomol Res.

[26] Geissbühler Y, Chaki P, Emidi B, Govella NJ, Shirima R, Mayagaya V (2007). Interdependence of domestic malaria prevention measures and mosquito-human interactions in urban Dar es Salaam, Tanzania. Malar J.

[27] Bayoh NM, Mathias DK, Odiere MR, Mutuku FM, Kamau L, Gimnig JE (2010). *Anopheles gambiae*: historical population decline associated with regional distribution of insecticide-treated bed nets in western Nyanza, Kenya. Malar J.

[28] Derua YA, Alifrangis M, Hosea KM, Meyrowitsch DW, Magesa SM, Pedersen EM, Simonsen PE (2012). Change in composition of the *An. gambiae* complex and its possible implications for the transmission of malaria and lymphatic filariasis in north-eastern Tanzania. Malar J.

[29] Mwangangi J, Mbogo C, Orindi B, Muturi E, Midega J, Nzovu J (2013). Shifts in malaria vector species composition and transmission dynamics along the Kenyan coast over the past 20 years. Malar J..

[30] Reddy MR, Overgaard HJ, Abaga S, Reddy VP, Caccone A, Michel A, Slotman MA (2011). Outdoor host seeking behavior of *Anopheles gambiae* mosquitoes following initiation of malaria vector control on Bioko Island, Equatorial Guinea. Malar J.

[31] Holt RA, Subramanian GM, Halpern A, Sutton GG (2002). The genome sequence of the malaria mosquito *An. gambiae*. Science.

[32] Mwangangi J, Muturi E, Muriu S, Nzovu J, Midega J, Mbog C (2013). The role of *Anopheles arabiensis* and *Anopheles coustani* in indoor and outdoor malaria transmission in Taveta District, Kenya. Parasit Vectors.

[33] Gillies MT, Coetzee M. A supplement to the anophelinae of African South of the Sahara (Afro Tropical region). Publ S Afr Inst Med Res, Johannesburg. 1987:55;1–43.

[34] Nyanjom SRG, Chen H, Gebre-Michael T, Bekele E, Shililu J, Githure J (2003). Population genetic structure of *Anopheles arabiensis* mosquitoes in Ethiopia and Eritrea. J Hered.

[35] Massebo F, Balkew M, Gebre-Michael T, Lindtjorn B (2013). Blood meal origins and insecticide susceptibility of *Anopheles arabiensis* from Chano in South-West Ethiopia. Parasit Vectors..

[36] Gerberg EJ (1970). Manual for mosquito rearing and experimental techniques. Am Mosq Control Assoc..

[37] Ponnuderai T, Lensen A, van Gemert V, Bensink MPE, Bolmer M, Meuwissen J (1989). Infectivity of cultured *Plasmodium falciparum* gametocytes to mosquitoes. Parasitol..

[38] Sinden R, Butcher G, Beetsma A (2007). Maintenance of the *Plasmodium berghei* life cycle. Methods Mol Med..

[39] Vlachou D, Kafatos F (2005). Complex interplay between mosquito positive and negative regulators of Plasmodium dev’t. Opinion Microbiol..

[40] Beier JC, Killeen GF, Githure JI (1999). Entomologic inoculation rates and *Plasmodium falciparum* malaria prevalence in Africa. Am J Trop Med Hyg..

[41] Lensen A, Van Druten J, Bolmer M, Van Gemert G, Eling W, Sauerwein R (1996). Measurement by membrane feeding of reduction in *Plasmodium falciparum* transmission induced by endemic sera. Trans R Soc Trop Med Hyg..

[42] Vaughan JA (2007). Population dynamics of *Plasmodium* sporogony. Trends Parasitol..

[43] Stone WJ, Eldering M, van Gemert GJ, Lanke KH, Grignard L, van de Vegte-Bolmer MG (2013). The relevance and applicability of oocyst prevalence as a read out for mosquito feeding assays. Sci Rep Parasite Biol..

[44] Ponnudurai T, Lensen A, Gemert G, Bolmer M, Meuwissen J (1991). Feeding behaviour and sporozoite ejection by infected *Anopheles stephensi*. Trans R Soc Trop Med Hyg..

[45] Rios-Velasquez C, Martins-Campos K, Simoes R, Izzo T, Santos E, Pessoa F (2013). Experimental *Plasmodium vivax* infection of key *Anopheles* species from the Brazilian Amazon. Malar J..

[46] Zhu G, Xia H, Zhou H, Lu F, Liu Y, Cao J (2013). Susceptibility of *Anopheles sinensis* to *Plasmodium vivax* in malarial outbreak areas of central China. Parasit Vectors.

[47] Bousema T, Collin J, Churcher T, Mulder B, Louis C, Geoffrey A (2011). Human immune responses that reduces the transmission of *Plasmodium falciparum* in African populations. Int Parasitol..

